# Predictive Roles of Baseline Stromal Tumor-Infiltrating Lymphocytes and Ki-67 in Pathologic Complete Response in an Early-Stage Triple-Negative Breast Cancer Prospective Trial

**DOI:** 10.3390/cancers15133275

**Published:** 2023-06-21

**Authors:** Nour Abuhadra, Ryan Sun, Clinton Yam, Gaiane M. Rauch, Qingqing Ding, Bora Lim, Alastair M. Thompson, Elizabeth A. Mittendorf, Beatriz E. Adrada, Senthil Damodaran, Kiran Virani, Jason White, Elizabeth Ravenberg, Jia Sun, Jaihee Choi, Rosalind Candelaria, Banu Arun, Naoto T. Ueno, Lumarie Santiago, Sadia Saleem, Sausan Abouharb, Rashmi K. Murthy, Nuhad Ibrahim, Aysegul Sahin, Vicente Valero, William Fraser Symmans, Jennifer K. Litton, Debu Tripathy, Stacy Moulder, Lei Huo

**Affiliations:** 1 Department of Breast Medical Oncology, The University of Texas MD Anderson Cancer Center, Houston, TX 77030, USA; abuhadn@mskcc.org (N.A.); cyam@mdanderson.org (C.Y.); sdamodaran@mdanderson.org (S.D.); jason.b.white812@gmail.com (J.W.); ravenberg_elizabeth@lilly.com (E.R.); barun@mdanderson.org (B.A.); nueno@cc.hawaii.edu (N.T.U.); ssaleem@mdanderson.org (S.S.); sabouharb@mdanderson.org (S.A.); rmurthy1@mdanderson.org (R.K.M.); nibrahim@mdanderson.org (N.I.); vvalero@mdanderson.org (V.V.); jlitton@mdanderson.org (J.K.L.); dtripathy@mdanderson.org (D.T.); moulder_stacy@lilly.com (S.M.); 2 Department of Biostatistics, The University of Texas MD Anderson Cancer Center, Houston, TX 77030, USA; rsun3@mdanderson.org (R.S.); jsun9@mdanderson.org (J.S.); 3 Division of Diagnostic Imaging, The University of Texas MD Anderson Cancer Center, Houston, TX 77030, USA; gmrauch@mdanderson.org (G.M.R.); beatriz.adrada@mdanderson.org (B.E.A.); rcandelaria@mdanderson.org (R.C.); lumarie.santiago@mdanderson.org (L.S.); 4 Department of Pathology, The University of Texas MD Anderson Cancer Center, Houston, TX 77030, USA; qqding@mdanderson.org (Q.D.); asahin@mdanderson.org (A.S.); fsymmans@mdanderson.org (W.F.S.); 5 Department of Oncology, Baylor College of Medicine, Houston, TX 77030, USA; bora.lim@bcm.edu; 6 Division of Surgical Oncology, Section of Breast Surgery, Baylor College of Medicine, Houston, TX 77030, USA; alastair.thompson@bcm.edu; 7 Division of Breast Surgery, Department of Surgery, Brigham and Women’s Hospital, Boston, MA 02115, USA; emittendorf@bwh.harvard.edu; 8 Division of Pathology and Laboratory Medicine, The University of Texas MD Anderson Cancer Center, Houston, TX 77030, USA; kvirani@mdanderson.org; 9 Department of Statistics, Rice University, Houston, TX 77005, USA; jc155@rice.edu

**Keywords:** triple-negative breast cancer, Ki-67, tumor-infiltrating lymphocytes, neoadjuvant chemotherapy, pathologic complete response, predictor

## Abstract

**Simple Summary:**

High stromal tumor-infiltrating lymphocytes (sTILs) are associated with improved pathologic complete response (pCR) in triple-negative breast cancer (TNBC). In this study of 408 patients enrolled in a prospective early-stage TNBC neoadjuvant chemotherapy trial, we aimed to identify clinicopathologic features that could be combined with sTILs to better predict pCR. Applying a training set and a testing set, we found that integrating high Ki-67 (cutoff > 35%) and high sTIL (cutoff ≥ 20%) in a model of computed response scores could predict a pCR rate of 65%. This model may refine the selection of early-stage TNBC patients for neoadjuvant clinical trials evaluating de-escalation strategies.

**Abstract:**

High stromal tumor-infiltrating lymphocytes (sTILs) are associated with improved pathologic complete response (pCR) in triple-negative breast cancer (TNBC). We hypothesize that integrating high sTILs and additional clinicopathologic features associated with pCR could enhance our ability to predict the group of patients on whom treatment de-escalation strategies could be tested. In this prospective early-stage TNBC neoadjuvant chemotherapy study, pretreatment biopsies from 408 patients were evaluated for their clinical and demographic features, as well as biomarkers including sTILs, Ki-67, PD-L1 and androgen receptor. Multivariate logistic regression models were developed to generate a computed response score to predict pCR. The pCR rate for the entire cohort was 41%. Recursive partitioning analysis identified ≥20% as the optimal cutoff for sTILs to denote 35% (143/408) of patients as having high sTILs, with a pCR rate of 59%, and 65% (265/408) of patients as having low sTILs, with a pCR rate of 31%. High Ki-67 (cutoff > 35%) was identified as the only predictor of pCR in addition to sTILs in the training set. This finding was verified in the testing set, where the highest computed response score encompassing both high sTILa and high Ki-67 predicted a pCR rate of 65%. Integrating Ki67 and sTIL may refine the selection of early stage TNBC patients for neoadjuvant clinical trials evaluating de-escalation strategies.

## 1. Introduction

The tumor immune microenvironment plays a fundamental role in the response to neoadjuvant chemotherapy (NACT) in triple-negative breast cancer (TNBC) [1,2,3,4]. Tumor-infiltrating lymphocytes (TILs) have emerged as a biomarker of the immune microenvironment, with important predictive and prognostic values in TNBC [5,6,7]. A pooled analysis of nine adjuvant clinical trials including 2148 TNBC patients demonstrated the strong prognostic role of sTILs in early-stage TNBC patients, in which patients with node-negative TNBC and ≥30% sTILs had a 3-year distant disease-free survival (dDFS) rate of 97% and overall survival (OS) rate of 99% [8]. In the neoadjuvant setting, multiple pooled analyses have confirmed that higher baseline TILs in pretreatment TNBC tumors are associated with higher rates of pCR, a well-established surrogate endpoint in neoadjuvant breast cancer trials [2,7,9,10,11,12]. In addition to the numerous studies that have validated the prognostic value of high sTILs in the context of systemic therapy, there are now also several retrospective studies demonstrating excellent clinical outcomes in early stage TNBC patients with high sTILs in the absence of chemotherapy [13,14]. Interestingly, a recent publication by Loi et al. demonstrated that incorporating TILs into the 8th Edition AJCC staging system has prognostic implications, and sTILs are able to up- and downstage traditional pathological staging. TILs represent the first immune biomarker to have an impact on traditional staging in breast cancer, highlighting the importance of integrating immune biomarkers into TNM staging and providing a model that could potentially be used in routine patient care as well as future clinical trial design [15]. 

Taken together, these data support the growing interest in utilizing sTILs to select TNBC patients for de-escalation strategies in appropriate clinical settings. In this study, we aimed to identify clinical and pathologic characteristics that are associated with improved pCR rates in early-stage TNBC treated with neoadjuvant anthracycline-based chemotherapy. We hypothesized that integrating high sTILs with additional clinical and pathologic features associated with pCR could strengthen the predictive power and enhance our ability to identify patients for whom de-escalation could be considered in the context of a clinical trial. 

## 2. Methods

### 2.1. Patient Population 

**Figure 1:**
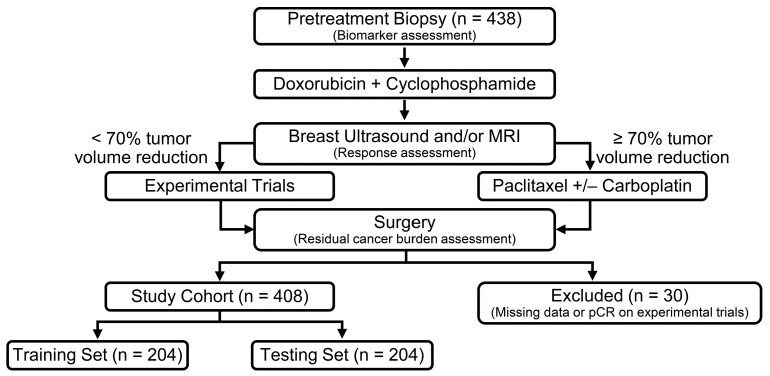
Trial design and study cohort. Biomarkers including sTIL, PD-L1, AR and Ki-67 were assessed in pretreatment tumors. Patients underwent imaging to assess treatment response after AC. Those who had a chemo-sensitive disease after 4 cycles of AC were recommended to proceed with standard taxane-based chemotherapy. Those who were predicted to be chemo-insensitive were offered therapy in clinical trials using targeted therapy in combination with chemotherapy based on the biomarker results of their tumors. The experimental trials were as follows: a phase II trial of neoadjuvant nab-paclitaxel and atezolizumab (NCT02530489); phase II trial of neoadjuvant liposomal doxorubicin, bevacizumab and everolimus (DAT) in TNBC insensitive to standard neoadjuvant chemo (NCT02456857); phase II trial of panitumumab, carboplatin and paclitaxel (PaCT) in localized TNBC insensitive to NACT (NCT02593175); and phase IIB neoadjuvant enzalutamide plus paclitaxel for AR+ TNBC (NCT02689427). AC, doxorubicin and cyclophosphamide; AR, androgen receptor; ARTEMIS, A Robust TNBC Evaluation fraMework to Improve Survival; NACT, neoadjuvant chemotherapy; pCR: pathologic complete response; sTIL: stromal tumor-infiltrating lymphocytes; TNBC: triple-negative breast cancer.

The patients included in this study were enrolled in the ARTEMIS trial (A Robust TNBC Evaluation fraMework to Improve Survival; NCT02276443), which was approved by the Institutional Review Board of the University of Texas MD Anderson Cancer Center. Written informed consent was obtained from the enrolled patients. In this trial, tumors from patients diagnosed with early-stage TNBC prior to receiving NACT were profiled, and a unique algorithm of diagnostic imaging and biomarkers was used to select the optimized targeted therapy trial for patients with chemotherapy-insensitive disease. The trial design was previously described [16]. Briefly, patients were first treated with 4 cycles of doxorubicin plus cyclophosphamide (AC) chemotherapy, followed by standard paclitaxel-based chemotherapy if they had chemotherapy-sensitive disease (≥70% tumor volumetric reduction on ultrasound after 4 cycles of AC) or were offered an experimental regimen with a standard chemotherapy backbone if they had chemotherapy-insensitive disease (<70% tumor volumetric reduction on ultrasound after 4 cycles of AC) before surgery. Patients diagnosed between October 2015 and October 2020 were included in this study. Thirty patients were excluded due to missing data or if they had a pathologic complete response (pCR) on experimental trials. The latter group were excluded because it was impossible to distinguish whether pCR was due to the effect of the targeted agents or due to the chemotherapy backbone. The remaining 408 patients constituted the study cohort (Figure 1).

### 2.2. Pathological Evaluation

Estrogen receptor and progesterone receptor negativity was defined as <10% staining of invasive carcinoma cells of any intensity according to immunohistochemistry. Human epidermal growth factor receptor 2 (HER2) negativity was defined according to the American Society of Clinical Oncology/College of American Pathologists HER2 testing guidelines [17,18,19,20]. Stromal TILs were assessed on hematoxylin- and eosin (H&E)-stained pretreatment (baseline) core biopsy slides based on the International TIL Working Group guidelines [6]. Percent stromal TILs was calculated as the area of the tumor stroma occupied by mononuclear inflammatory cells divided by the total tumor stromal area. Stromal TILs were evaluated by central pathology review and recorded in increments of 10%, with values of 5% and above rounded up to the next-highest increment (Supplementary Figure S1A,B). pCR was defined as the absence of invasive cancer within the breast and regional lymph nodes (ypT0/is ypN0), and residual disease was defined as the lack of pCR. The residual cancer burden (RCB) index and class were calculated according to previously published methods based on the histopathological features of the residual disease in specimens after NACT [21,22].

### 2.3. Immunohistochemistry

Immunohistochemical (IHC) staining was performed on unstained 4 μm thick tissue sections from a representative paraffin block of the pretreatment tumor in each case. The slides were incubated at 60 °C for 25 min. IHC staining for PD-L1 was performed using the FDA-approved PD-L1 IHC 22C3 pharmDx kit (Dako, Carpinteria, CA, USA) on a Dako AutostainerLink 48, according to the manufacturer’s instructions. IHC staining for the androgen receptor (AR) and Ki-67 was performed using the polymeric biotin-free horseradish peroxidase method with a Leica Microsystems Bond III autostainer (Leica Microsystems, Buffalo Grove, IL, USA). Ki-67 was performed if the results were not collected from the diagnostic pathology reports. For AR, following heat-induced epitope retrieval with citrate buffer for 25 min at 100 °C, the slides were incubated with mouse monoclonal antibody to AR (clone AR441, Dako; 1:30). For Ki-67, following heat-induced epitope retrieval with Tris-EDTA buffer for 20 min at 100 °C, the slides were incubated with mouse monoclonal antibody to Ki-67 (clone MIB-1, Dako; 1:100). The Refine Polymer Detection kit was used to detect bound antibody, with 3,3-diaminobenzidine serving as the chromogen (Leica Microsystems). The slides were counterstained with Mayer’s hematoxylin. The results were evaluated with known positive and negative tissue controls. 

For PD-L1, the Combined Positive Score (CPS) was calculated as the number of PD-L1-stained cells (viable invasive tumor cells showing membranous staining of any intensity; lymphocytes and macrophages in the tumor area showing membranous or cytoplasmic staining of any intensity) divided by the total number of viable invasive tumor cells, multiplied by 100. For AR, the percentage and intensity of any nuclear staining of the tumor cells were recorded. For Ki-67, the percentage of nuclear staining of any intensity in the tumor cells was recorded (Supplementary Figure S1C,D).

### 2.4. Statistical Analysis 

Training and testing sets were created through randomization, stratified based on sTIL status and the date of enrollment. Briefly, patients were first placed in the order of the date of enrollment in the following sTIL groups: <5%, 10%, 20%, 30%, 40–50%, 60–90%. The higher-sTIL categories were grouped together because each had a small number (<20) of patients. Next, every other patient in each sTIL group was selected into a training set. The training sets from all the sTIL groups were then incorporated into the final training set (*n* = 204). The remaining patients formed the testing set (*n* = 204). A comparison of the two sets for the low-sTIL and high-sTIL patients is shown in Supplementary Table S1. For the model selection, we utilized recursive partitioning analysis, which is a random-forest-based algorithm that splits data into subgroups based on pCR status. Table 1 shows the variables included in the analysis, with the continuous variables divided into high and low groups based on the best cutoffs in association with pCR identified in the analysis. This method optimally selects among multiple variables to find the models that best predict pCR. In the training set, the results of the recursive partitioning were combined with clinical expertise to determine the variables selected for inclusion in a final logistic regression model. A risk score was created from this final logistic regression model, with weights given to the covariates according to their regression coefficients. The risk score was then applied to stratify the subjects in the testing set. Mean pCR rates along with 95% exact confidence intervals were calculated for each stratum in the testing set. We assessed the differences in clinicopathologic characteristics between high and low TILs using the chi-squared test and Fisher’s exact test. For all statistical analyses, *p*-values of 0.05 or less were considered significant. 

## 3. Results

### 3.1. Patient Baseline Characteristics

**Figure 2:**
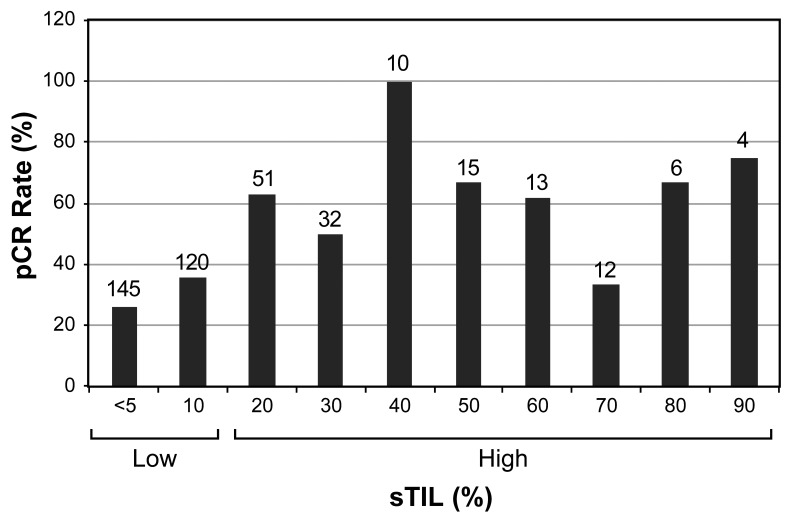
pCR rates in sTIL groups among 408 patients. The total number of patients in each sTIL group is shown at the top of the bars. Recursive partitioning analysis identified ≥20% as the value for defining high sTIL in association with pCR. pCR, pathologic complete response; sTIL, stromal tumor-infiltrating lymphocytes.

Patient characteristics of the 408 patients with early-stage TNBC included in this study are shown in Table 1. In the total population, the majority of patients had clinical stage II disease (70%), were node-negative (60%) and were of high histologic grade (87%). The majority of the patients also had high Ki-67 (82%) and were PD-L1 positive (65%). The pretreatment core biopsies were evaluated for sTILs on the H&E sections. Through recursive partitioning analysis, 20% was identified as the optimal cutoff to define high sTILs (≥20%) in association with pCR (Figure 2). Using this cutoff, 265 patients were classified as low-sTIL, and 143 patients as high-sTIL. 

### 3.2. Predictors of pCR in Total, Low-sTIL and High-sTIL Patient Populations 

Of the 408 patients included in this study, 166 patients experienced a pCR (41%). Among the clinicopathologic variables, sTIL ≥ 20% (*p* = 0.0004) and Ki-67 > 35% (*p* = 0.001) emerged as being independently and significantly associated with an increased likelihood of pCR in the training set (*n* = 204). When applied to the testing set (*n* = 204), these variables remained significantly associated with pCR (sTIL, *p* = 0.0004; Ki-67, *p* = 0.003). Based on their similar coefficients in the logistic regression model, each variable was given an equivalent weight in the computed response score (CRS) generated using the testing set (Table 2), with each being worth one point (highest CRS = 2 points). In patients with high sTIL and high Ki-67 (CRS 2, *n* = 65), the pCR rate was 65% (95% CI 52–76%). 

Next, recognizing that the predictors of response may differ between tumors with low sTILs and those with high sTILs, we separated the entire cohort into low- versus high-sTIL patient populations to investigate which variables may be associated with pCR. As shown in Table 1, high-sTIL patients were more likely to have T1 or T2 disease, a higher Ki-67 proliferation index, and PD-L1 positivity compared with low-sTIL patients. With multivariate cox regression analysis in the low-sTIL patient population, no clinically meaningful variables were identified as good predictors of pCR. Interestingly, in the high-sTIL patient population, Ki-67 > 35% emerged as a single predictor of pCR (*p* = 0.0003).

## 4. Discussion

In this study, we assessed clinical and pathologic predictors of pCR in patients with early stage TNBC treated with NACT in a prospective trial. Historically, the reported rates of pCR in patients with early stage TNBC treated with anthracycline-based NACT have ranged between 35 and 50% [23,24,25,26,27]. Our results show that in this setting, the combination of high sTILs (≥20%) and high Ki-67 (>35%) predict a higher pCR rate of 65%. In addition to the many studies in the literature confirming the predictive value of high sTILs, some have also identified Ki-67 as a predictive marker in the NACT of TNBC [28,29,30]. One meta-analysis including seven TNBC studies showed that high Ki-67 was a predictor of higher pCR rates in four of them, among which one study demonstrated Ki-67 as an independent predictor [29]. In another meta-analysis, seven of fourteen neoadjuvant TNBC studies showed a significant direct association between Ki-67 and pCR [30]. However, Ki-67 was included in only a small number of TNBC cohorts to examine its role as a predictor for pCR along with sTILs, and most of the cohorts were retrospectively collected, with less than 200 patients [31,32,33,34,35,36]. Although Ki-67 did not emerge as a significant predictor independent of sTILs in most of those studies, one retrospective study of 166 patients with TNBC identified Ki-67 as a predictor in addition to sTILs [33]. In our cohort, Ki-67 emerged as the only predictor for pCR in addition to sTILs when a training set and a testing set were analyzed. This predictive value of Ki-67 was confirmed in the high-sTIL group when we analyzed the high-sTIL and low-sTIL patients separately. To our knowledge, our study represents the largest prospectively collected single cohort of early-stage TNBC used to investigate the roles of clinicopathologic factors in predicting pCR. It is interesting to note that in our results, the improvement obtained by adding Ki-67 to sTILs in the prediction model was mild, being approximately 6% compared with high sTILs alone. It is possible that such an effect can only be detected when a relatively large cohort is tested, which may explain the negative association found in other smaller studies. 

The patients in this study were enrolled and treated with anthracycline-based chemotherapy prior to the 2021 FDA approval of neoadjuvant and adjuvant pembrolizumab following results from the landmark trial KEYNOTE-522; however, the implications remain relevant [37]. The combination of high sTILs and high Ki-67 identifies a subset of patients with an excellent response to NACT (65%), similar to the pCR rates reported with the KEYNOTE-522 regimen (64.8%) but without the additional toxicity of immunotherapy. It is worth noting that immune-mediated adverse events occurred in 43.6% of patients in the pembrolizumab arm. A limitation of our study is that it was conducted prior to the approval of checkpoint inhibitors for early-stage TNBC; additional analyses are needed to evaluate the association between high sTILs, Ki-67 and pCR in patients receiving neoadjuvant immunotherapy. While this study was conducted using prospectively collected data in a clinical trial setting, an external validation cohort is needed to confirm these findings. If confirmed, baseline sTILs and Ki-67 may allow us to identify patients for whom immune checkpoint blockade can be omitted and could be considered as a patient selection strategy for de-escalation trials. Further studies are required to identify clinical and pathologic predictors of the response to neoadjuvant chemoimmunotherapy. 

Another limitation of this study is that nearly all the patients received combination anthracycline and taxane chemotherapy; therefore, we were unable to assess whether specific chemotherapy regimens were associated with different patterns of immune response, other clinicopathologic factors and the ultimate pathologic outcomes. Biomarker evaluation including sTIL, AR, Ki-67 and PD-L1 was performed on core biopsy materials as a representation of the entire tumor; therefore, limited sampling is an intrinsic limitation of this neoadjuvant study. While Ki-67 was identified as a strong predictor in the high-sTIL group, thus confirming our results using the training and testing sets, our study did not identify any clinically meaningful factors that could predict pCR in the low-sTIL group. This finding is intriguing, considering that 31% of patients in the low-sTIL group achieved pCR. Further work is needed using alternative analytic methods (e.g., genomics, transcriptomics and proteomics, as well as morphologic analysis using artificial intelligence tools) to identify predictors of resistance and/or response in this population. The early detection of treatment response during NACT may also facilitate the proper triaging of patients, including low-sTIL patients, for further therapy.

In conclusion, we demonstrated that integrating Ki-67 with sTILs provides independent and additional information beyond baseline sTILs in the prediction of pCR in patients with early-stage TNBC. The incorporation of Ki67 (>35%) and sTILs (≥20%) may refine the selection of this group of patients for neoadjuvant clinical trials evaluating de-escalation strategies upon further validation.

## Figures and Tables

**Table 1:** Patient clinicopathologic characteristics.

	Overall	Low sTILs (<20%)	High sTILs (≥20%)	*p*-Value
**Total patients**	408	265	143	NA
**Median age at diagnosis—years (range)**	51 (23–77)	52 (24–77)	49 (23–77)	0.17
**BMI at diagnosis—*n* (%)**				
Low (<25)	111 (27)	67 (25)	44 (31)	0.23
High ≥25	297 (73)	198 (75)	99 (69)	
**Tumor Stage—*n* (%)**				
T1	66 (16)	37 (14)	29 (20)	
T2	273 (67)	168 (63)	105 (73)	**<0.001**
T3	49 (12)	42 (16)	7 (5)	
T4	20 (5)	18 (7)	2 (1)	
**Nodal Stage—*n* (%)**				
N0	246 (60)	165 (62)	81 (57)	
N1	98 (24)	56 (21)	42 (29)	**0.01**
N2	10 (2)	3 (2)	7 (5)	
N3	54 (13)	41 (15)	13 (9)	
**Clinical TNM Stage—*n* (%)**				
I	41 (10)	28 (11)	13 (9)	
II	284 (70)	177 (67)	107 (75)	0.22
III	83 (20)	60 (22)	23 (16)	
**Histologic Grade—*n* (%)**				
1	2 (1)	2 (1)	0	
2	51 (12)	43 (16)	8 (5)	**0.002**
3	355 (87)	220 (83)	135 (95)	
**Histologic Type—*n* (%)**				
Invasive Ductal	342 (84)	213 (80)	129 (90)	
Invasive Lobular	3 (1)	2 (1)	1 (1)	
Metaplastic	44 (11)	34 (13)	10 (7)	0.051
Other *	19 (4)	16 (6)	3 (2)	
**Ki-67—*n* (%)**				
Low (≤35%)	72 (18)	58 (22)	14 (10)	
High (>35%)	336 (82)	207 (78)	129 (90)	**0.003**
**Androgen Receptor Expression—*n* (%)**				
Low (<10%)	309 (76)	203 (77)	106 (74)	0.63
High (≥10%)	99 (24)	62 (23)	37 (26)	
**PD-L1 Expression (CPS)—*n* (%)**				
Negative (0)	144 (35)	132 (50)	12 (8)	**<0.001**
Positive (>0)	264 (65)	133 (50)	131 (92)	
**Pathologic Response—*n* (%)**				
pCR/RCB-0	166 (41)	81 (31)	85 (59)	
RCB I	61 (15)	42 (16)	19 (13)	**<0.001**
RCB-II	140 (34)	112 (42)	28 (20)	
RCB-III	41 (10)	30 (11)	11 (8)	

* Includes invasive mammary, apocrine, epithelioid, mixed and neuroendocrine.

**Table 2:** Computed response score (testing set, *n* = 204).

Computed Response Score	N	No. pCR	% pCR	95% CI (%)
0	28	5	18	6–37
1	111	35	32	23–41
2	65	42	65	52–76

## Data Availability

The data that support the findings of this study are available from the corresponding author upon reasonable request.

## Supplementary Materials

The following supporting information can be downloaded at: https://www.mdpi.com/article/10.3390/cancers15133275/s1, Figure S1. Photomicrographs of representative tumors. Table S1. Comparison of the characteristics of the training set and testing set.
