# Supplementary material for: Predictive Roles of Baseline Stromal Tumor-Infiltrating Lymphocytes and Ki-67 in Pathologic Complete Response in an Early-Stage Triple-Negative Breast Cancer Prospective Trial
Source: Cancers (Basel). 2023 Jun 21;15(13):3275. doi: 10.3390/cancers15133275 (PMC10339918; doi:10.3390/cancers15133275)
Supplement: Supplementary file 1 [file cancers-15-03275-s001.zip › cancers-2436535-supplementary.pdf]

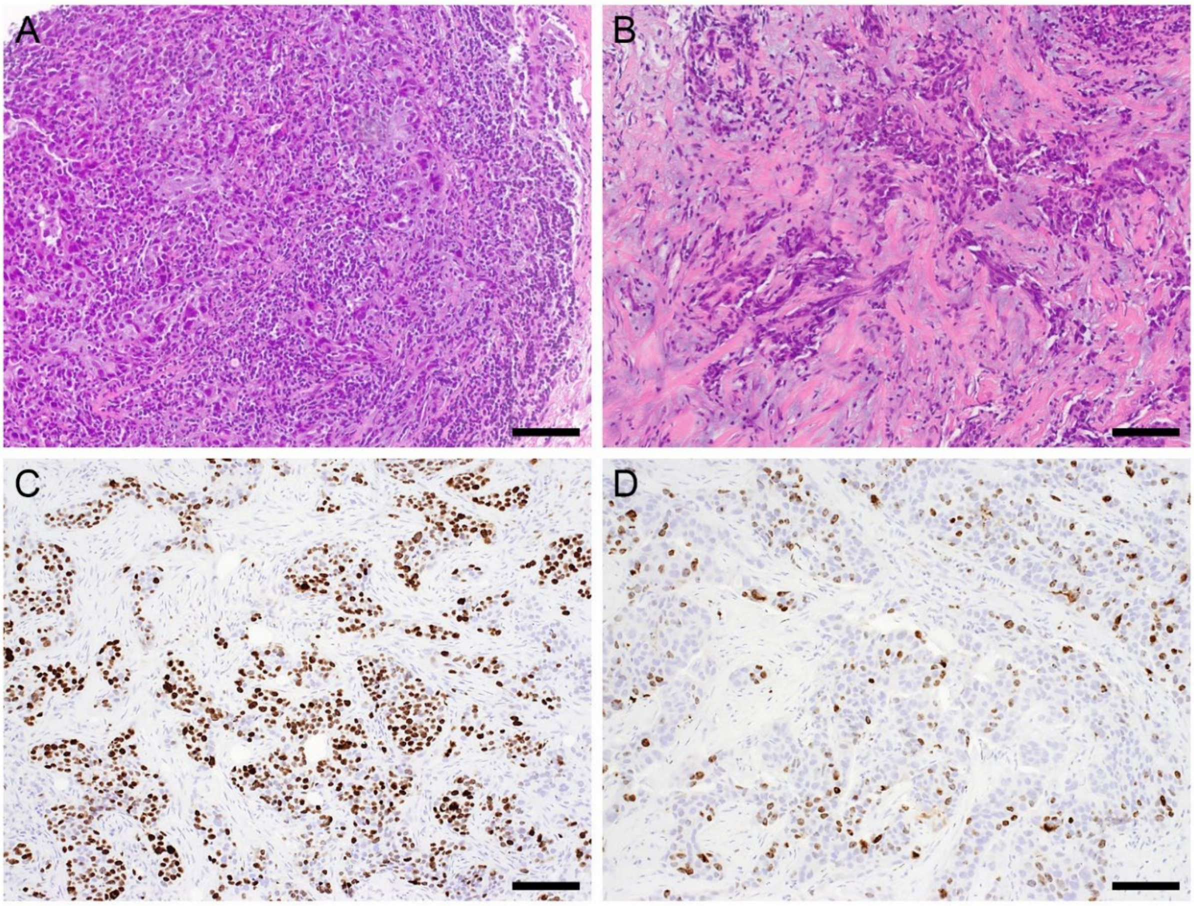

Supplementary Figure S1. Photomicrographs of representative tumors. (A) A tumor with high sTIL(hematoxylin and eosin stain). (B) A tumor with low sTIL (hematoxylin and eosin stain). (C) A tumor with high Ki-67 (immunohistochemistry stain). (D) A tumor with low Ki-67 (immunohistochemistry stain). Scale bar, 100 µm.
sTIL, stromal tumor-infiltrating lymphocytes.

Supplementary Table S1. Comparison of the characteristics of the training set and testing set

	Low sTIL			High sTIL		
	Training set* (n=133) Mean (SD)	Testing set (n=132) Mean (SD)	p-value	Training set (n=71) Mean (SD)	Testing set (n=72) Mean (SD)	p-value
**STIL**	6.71 (3.00)	6.73 (3.00)	0.956	39.01 (20.99)	39.17 (20.47)	0.965
**Age at diagnosis (years)**	52.47 (11.33)	51.05 (12.40)	0.331	50.13 (11.64)	49.99 (11.92)	0.943
**BMI at diagnosis**	31.79 (12.31)	29.85 (7.18)	0.119	29.80 (6.89)	27.57 (5.52)	0.034
**Tumor Stage**	2.08 (0.70)	2.23 (0.77)	0.079	1.87 (0.51)	1.88 (0.58)	0.985
**Nodal Stage**	0.68 (1.10)	0.71 (1.07)	0.834	0.72 (1.02)	0.61 (0.85)	0.495
**Clinical TNM Stage**	2.08 (0.59)	2.16 (0.54)	0.272	2.11 (0.55)	2.03 (0.44)	0.310
**Histologic Grade**	2.83 (0.38)	2.82 (0.42)	0.858	2.93 (0.26)	2.96 (0.20)	0.458
**Histologic Type#**	0.10 (0.30)	0.16 (0.37)	0.136	0.00 (0.00)	0.14 (0.35)	0.001
**Ki-67**	63.23 (26.71)	60.75 (25.48)	0.441	70.38 (23.12)	70.21 (22.19)	0.964
**Androgen Receptor**	9.89 (22.30)	10.06 (21.82)	0.950	11.88 (23.17)	8.03 (17.74)	0.266
**PD-L1 (CPS)**	3.08 (8.46)	3.70 (11.44)	0.615	20.31 (29.06)	18.85 (25.33)	0.749
**pCR**	0.31 (0.46)	0.30 (0.46)	0.927	0.59 (0.50)	0.58 (0.50)	0.921

*The training sets from the low sTIL and high sTIL groups were combined to form the training set of the entire cohort (N=204). The testing sets from the low sTIL and high sTIL groups were combined to form the testing set of the entire cohort (N=204).
#Some variables were coded numerically for comparison.
STIL: stromal tumor-infiltrating lymphocytes.
